# The Role of Artificial Intelligence and Machine Learning Applications in Emergency Surgery: A Systematic Review of Diagnostic Accuracy and Clinical Outcomes

**DOI:** 10.7759/cureus.85386

**Published:** 2025-06-05

**Authors:** Safa Baqar, Adel S Hamed, Islam Elbreki, Tarig Mohamed, Bakhtawar Awan, Mohamed Elsaigh

**Affiliations:** 1 General Surgery, North Middlesex University Hospital, London, GBR; 2 Colorectal Surgery, Aneurin Bevan University Health Board, Caerleon, GBR; 3 Faculty of Medicine and Surgery, Benghazi University, Benghazi, LBY; 4 Anesthesiology, Barnet Hospital, Royal Free London NHS Foundation Trust, London, GBR; 5 Gastroenterology, Royal Devon and Exeter Hospital, Royal Devon University Healthcare NHS Foundation Trust, Devon, GBR; 6 General Surgery, London North West University Healthcare NHS Trust, Harrow, GBR; 7 General and Emergency Surgery, Royal Cornwall Hospital, Truro, GBR

**Keywords:** artificial intelligence, diagnostic accuracy, emergency surgery, machine learning, systematic review

## Abstract

Artificial intelligence (AI) refers to computer systems' ability to perform tasks requiring human intelligence. In recent years, AI has rapidly evolved in various fields, including the medical field. The integration of AI into emergency surgical care represents a significant advancement in modern medicine. This field has developed rapidly, particularly since the mid-2010s. The advancement in AI-assisted emergency surgery is built upon several technological pillars, such as deep learning, natural language processing for rapid medical record analysis, and integration with existing hospital information systems. We aim to evaluate the effectiveness of machine learning in identifying emergency patients and the effectiveness of AI methods in diagnosing them compared to conventional methods. We also aim to assess AI's capability in predicting complications and the need for surgical intervention.

The systematic review included English-language research papers published between 2015 and 2025 focusing on human studies. Two independent reviewers analyzed articles following the Preferred Reporting Items for Systematic Reviews and Meta-Analyses (PRISMA) guidelines, screening titles from five significant databases (PubMed, Web of Science, Scopus, Cochrane, and Embase). The final selection process ensured all included studies aligned with the primary research question examining machine learning's impact on emergency care accuracy and efficiency.

This systematic review identified 19 eligible studies from an initial pool of 2791 publications. The results showed the significance of the application of AI across five key areas of emergency surgery: appendicitis management (five studies), emergency abdominal surgery risk assessment (five studies), acute abdominal pain and triage (two studies), bowel obstruction (four studies), and acute conditions of the gallbladder and mesenteric vessels (three studies). Machine learning models demonstrated promising accuracy rates compared to conventional methods in all the different aspects.

This systematic review highlights the promising impact of AI and machine learning across emergency surgery domains. The models demonstrated remarkable accuracy (72-98%) across various applications, from appendicitis diagnosis to cholecystitis detection. Most notably, AI tools showed superior performance in acute abdominal pain triage and risk assessment compared to conventional methods, suggesting their potential to enhance clinical decision-making in emergency surgical settings.

## Introduction and background

Artificial intelligence (AI) refers to the capability of computers to perform tasks that require human intelligence [1]. The use of AI in medicine is expanding with a significant possibility to transform patient care by progressing personalized medicine and personalizing treatments for individual patients [2]. Machine learning (ML) is a subset of AI that enables systems to make predictions or decisions without explicit programming [3]. The principle of ML is to inherit properties of data and past experiences to develop models that can accurately predict outcomes for new instances based on patterns learned from training datasets [4].

A proceeded subset of ML called deep learning (DL) uses artificial neural networks (ANN) to analyze sophisticated data patterns and effectively process unstructured data types, including images, audio, and text [5]. In addition, DL can learn and make decisions autonomously, mimicking the functional processes of the human brain based on the ANN [6]. ANN was invented to mimic the human brain's neuronal interconnections. The node within the ANN is designed to do a similar function to the neuron as follows: receive inputs from and donate outputs to surrounding nodes [7]. That makes it similar to the neural network of the human central nervous system. A more advanced subset of ANN is the convolutional neural network (CNN), which can process more complicated data, such as languages and audio, in addition to spatial perception. Furthermore, CNN can use both symptoms and medical imaging to make a professional diagnosis [8].

AI was described for the first time in 1950 [9]. Despite the rapid advancements of AI in engineering, progress in the medical field has been relatively slow, as the first application for AI in clinical medicine was in the 1970s [10]. INTERNIST-1 was the first AI-based medical consultant that uses algorithms to help physicians diagnose diseases based on input symptoms [11]. Also, in the 1970s, the MYCIN system was designed to help prescribe the optimal antibiotics in cases of infections based on input criteria [12]. INTERNIST-1 represented a pioneering advancement in applying AI to medical diagnosis. It demonstrated the viability of AI as a tool to support and enhance clinical decision-making. This system laid the foundation for subsequent innovations, such as DXplain and other modern AI-based diagnostic applications [11]. DXplain was the second chief development that appeared in the 1980s. It built upon the foundation of INTERNIST-1; by increasing the scope of clinical diagnoses, it can derive and provide up-to-date medical information for healthcare providers [9]. ANN was first used in clinical medicine in 1995, and since its introduction into the medical field, it has become the cornerstone in the development of AI in medicine [13].

In 2007, IBM developed a question-answering system (Watson) that demonstrated its capabilities by surpassing top contestants and champions on the television quiz show Jeopardy. Watson utilized DeepQA, which is a language-processing framework that enabled it to analyze data from various contexts and extract relevant information from multiple sources to generate accurate responses [14,15]. This advancement expanded AI applications in healthcare as input data was no longer restricted to symptoms and outputs could extend beyond conventional clinical diagnoses. In 2017, Watson successfully identified RNA-binding proteins associated with amyotrophic lateral sclerosis (ALS). Additionally, novel AI systems were developed to enhance patient care across various domains. One such example is Pharmbot, introduced in 2015, designed to educate patients and their families about medications and treatment protocols [9]. Previously, the robotic assistance was evaluated in different studies without a clear focus on the type of system used, so we aim to shed more light on the different types of AI systems or the way of the assistance provided by AI [16].

In this systematic review, we aim to evaluate whether ML approaches can improve the accuracy and efficiency of identifying patients who require emergency surgery, especially when compared to conventional methods. In addition to the prediction of complications and the need for surgical intervention in patients, we also aim to estimate the degree of superiority AI would provide compared to conventional ways.

## Review

Materials and methods

This review has been conducted in accordance with the guidelines of the Preferred Reporting Items for Systematic Reviews and Meta-Analyses (PRISMA) [17].

Review Question

The goal of this systematic review was to answer the following question: "Can ML approaches enhance the accuracy and efficiency of identifying patients requiring emergency care, predicting complications, and determining the need for surgical intervention compared to conventional methods?"

Literature Search

The literature search was done in five different electronic databases (PubMed, Web of Science, Scopus, Cochrane, and Embase) with the search strategy of ("artificial intelligence" OR "AI" OR "machine learning" OR "deep learning" OR "computer-assisted diagnosis" OR "AI-driven decision-making" OR "AI-based diagnostics" OR "AI-powered tools") AND (("emergency" OR "acute care" OR "trauma" OR "critical care" OR "damage control") AND (surger*)) in taking into consideration the question of our review.

The search was conducted in January 2025. Relevant articles were identified and selected by reviewers through an initial screening of titles and introductions. Full-text articles were retrieved for those meeting the eligibility criteria. Subsequently, study design and research data were extracted from each selected article. Articles that did not align with the main focus of our review were reassessed, and a final decision was made regarding their relevance.

Inclusion Criteria

Any research paper published in the English language, articles published for a period of 10 years from 2015 to 2025, and studies conducted on humans only were included in the study.

Exclusion Criteria

Excluded were non-English publications, conference abstracts without full-text availability, duplicate publications or overlapping datasets, case reports and case series, studies conducted on animals, and studies not applying AI or ML within their criteria.

Critical Appraisal

In accordance with the eligibility criteria and PRISMA guidelines, the included articles were independently analyzed by both reviewers. Any reviewer discrepancies were resolved through discussions between the two authors until a consensus was reached.

Quality Assessment

We estimated the quality of the included studies using the Quality Assessment of Diagnostic Accuracy Studies-2 (QUADAS-2) tool. The tool is validated to evaluate the quality of diagnostic accuracy studies and is widely recommended for systematic reviews in this field. The QUADAS-2 tool evaluates study risk of bias across four key domains: (1) patient selection, (2) index test, (3) reference standard, and (4) flow and timing. Additionally, another three domains are evaluated for concerns regarding applicability to the review question.

Results

**Figure 1:**
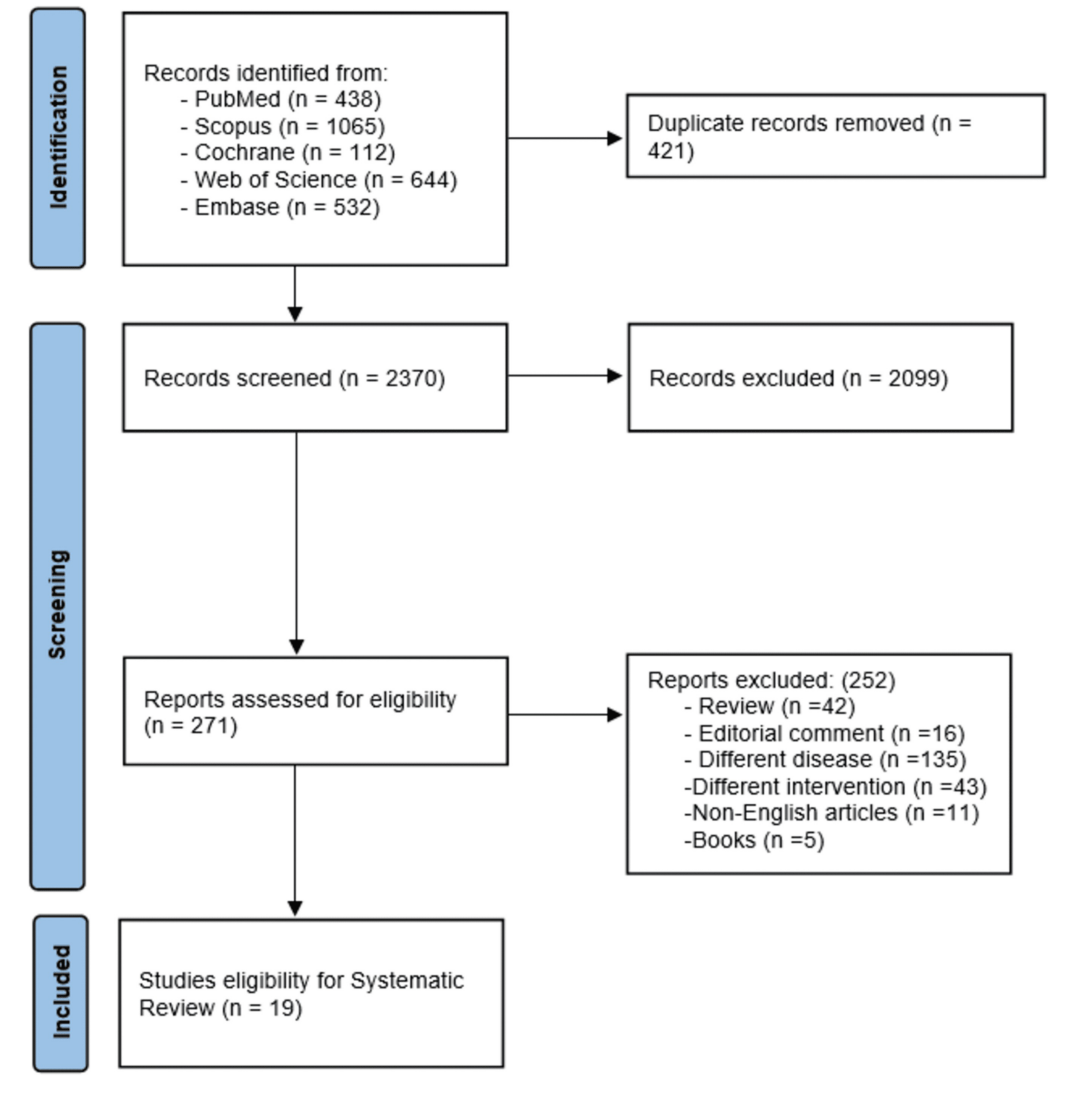
PRISMA flow diagram PRISMA: Preferred Reporting Items for Systematic Reviews and Meta-Analyses

Using the search strategy in the five electronic libraries, the search strategy yielded a total of 2791 studies, of which 2520 articles were either irrelevant or duplicated. Of the relevant 271 studies, 19 met the eligibility criteria and were included in this systematic review [18-36]. The flowchart of the strategy search for this systematic review has been summarized and presented in Figure 1.

Baseline and Summary of the Included Studies

The studies were conducted across various countries, with the United States, China, and Turkey emerging as the leading countries conducting multiple studies on AI applications in emergency surgery, with most studies focusing on common acute abdominal conditions such as appendicitis, bowel obstruction, and colorectal emergencies. Random forest and logistic regression consistently appear as the most frequently utilized AI models across studies, regardless of geographic location, while the primary outcomes measured throughout these investigations include mortality prediction, complication rates, and diagnostic accuracy. All the detailed data of baseline and summary characteristics of the included studies are presented in Table 1.

**Table 1:** Baseline and summary of the included studies AI: artificial intelligence; ML: machine learning; RF: random forest; SVM: support vector machine; GBDT: gradient boosting decision trees; MLP: multilayer perceptron; LR: logistic regression; GBM: gradient boosting machine; XGBoost: eXtreme gradient boosting; DNN: deep neural network; SSI: surgical site infection; NB: naive Bayes; POTTER: Predictive Optimal Trees in Emergency Surgery Risk; OCT: optimal classification tree; AUC: area under the curve; ANN: artificial neural network; KNN: K-nearest neighbor; DT: decision tree; BRF: balanced random forest; AUC-ROC: area under the receiver operating characteristic curve; PRF: platelet-rich fibrin; ccfDNA: circulating cell-free DNA; CNN: convolutional neural network; HAS: Heidelberg appendicitis score; PPV: positive predictive value; NPV: negative predictive value; CART: classification and regression tree; XGBC: XGBoost classifier

Study ID and year	Study design	Study population	Study aspect	Main outcomes	AI used	Country
Akabane et al. 2023 [23]	A retrospective cohort	8792	Patients undergoing emergency colorectal surgery	In-hospital mortality for patients who underwent emergency colorectal surgery, colorectal cancer presence, use of laparoscopy	Logistic regression and three supervised ML models: RF, GBDT, and MLP	Japan
Akmese et al. 2020 [18]	A retrospective cohort	428	Diagnosis of patients with early acute appendicitis	Accurate prediction of appendicitis surgery necessity, model prediction accuracy/performance metrics	They used 5 ML models: gradient boosted trees, RF, neural network, LR, and SVM	Turkey
Chai et al. 2024 [28]	A retrospective cohort	38,214	Abdominal pain surgery patients	Postoperative mortality and postoperative complications	They developed 5 ML models: light GBM, XGBoost, DNN, RF, and LR	China
Chen et al. 2024 [24]	A retrospective cohort	10,993	Patients undergoing emergency general surgery	Predicted five major postoperative events: mortality, pneumonia, SSI, thrombosis, and mechanical ventilation >48 hours	RF, SVM, NB, XGBoost, LR	China
El Hechi et al. 2021 [25]	A retrospective cohort	59,955	Emergency general surgery patients	Main outcomes: 30-day mortality, overall morbidity, and complications	POTTER calculator using OCT methodology, ML-based risk prediction tool, non-linear prediction model, and interactive smartphone application interface	United States
Gan et al. 2024 [29]	A retrospective cohort	2412	Patients who visited the emergency department with acute abdominal pain	Identification of 11 significant clinical indicators associated with acute abdominal pain severity, performance metrics of prediction models (accuracy, AUC, F1 score, recall), best-performing model selection and optimization	Eight models tested: ANN, LR, KNN, SVM, kernel SVM, DT, RF, and XGBoost	China
Ghomrawi et al. 2023 [19]	A retrospective cohort	161	Children with complicated and simple appendicitis	Detection of abnormal recovery events 2 days before occurrence, detection rate of 83% for complicated appendicitis cases, detection rate of 70% for simple appendicitis cases, performance metrics	BRF classifier, adaptive boosting, XGBoost, RUSBoost, balanced bagging, and easy ensemble	United States
Hadaya et al. 2022 [26]	A retrospective cohort	1,092,011	Patients with acute respiratory failure following emergency general surgery operations	Prediction of postoperative respiratory failure, model performance metrics: AUC-ROC, precision-recall curves, calibration, feature importance, impact of PRF on mortality, length of stay, and costs	AI/ML used: primary model: XGBoost, LR	United States
Harmantepe et al. 2023 [20]	A retrospective cohort	345	Diagnostic prediction of acute appendicitis	Voting classifier accuracy, sensitivity, and specificity	LR, SVM, KNN, neural networks, and an ensemble algorithm using the voting method	Turkey
Harmantepe et al. 2024 [34]	A retrospective cohort	122	Patients diagnosed with acute mesenteric ischemia	Mortality prediction in acute mesenteric ischemia and using serum-based laboratory results, sensitivity, specificity, and AUC	LR, RF, KNN, multilayer perceptron, support vector classifier	Turkey
Mazzotta et al. 2024 [30]	A retrospective cohort	99	Patients undergoing emergency surgery for bowel obstruction	Major complication rate, major complications, identified C-reactive protein, and cancer-related obstruction	KNN, XGBoost, and LR	Italy
Nishiwaki et al. 2017 [31]	A retrospective cohort	19	Patients with small bowel strangulation	Demonstrated ML can detect small bowel strangulation based on ccfDNA concentration, evaluation of accuracy, precision, and recall	SVM, DT, and RF	Japan
Oh et al. 2023 [35]	A retrospective cohort	578	High-risk acute small bowel obstruction patients	Accuracy, sensitivity, specificity, PPV, NPV, and AUC	3D CNN (incorporating dual-branch architecture with depth retention pooling), WideResNet, DenseNet, EfficientNet	South Korea
Okuda et al. 2022 [32]	A retrospective cohort	154	Diagnosing patients with gangrenous cholecystitis on computed tomography	Accuracy, sensitivity, specificity, and AUC	CNN, Xception architecture, and trained on ImageNet pre-trained data	Japan
Qiu et al. 2021 [33]	A retrospective cohort	526	Patients with intestinal obstruction, whether with or without intestinal necrosis	Outcomes on two-layer models estimating accuracy, sensitivity, specificity, and the Matthews correlation coefficient	DNN, SVM, and RF	China
Reismann et al. 2019 [21]	A retrospective cohort	590	Pediatrics with acute appendicitis, whether in diagnosis or classification	Accuracy, sensitivity, specificity, and AUC for diagnosing appendicitis or differentiating complicated vs. uncomplicated appendicitis	Linear LR model	Germany
Saboorifar et al. 2024 [36]	A retrospective cohort	534	Patient data with abdominal pain lasting one week or less	Accuracy, sensitivity, specificity, AUC-ROC, and Brier score	SVM algorithm using the radial basis kernel function	Iran
Stiel et al. 2020 [22]	A retrospective cohort	463	Predicting appendicitis in children	Modified HAS performance and AI score performance in terms of sensitivity, specificity, PPV, and NPV	RF algorithm, CART analysis	Germany
Xue et al. 2021 [27]	A secondary data analysis study	926	Prediction of pulmonary complications after emergency gastrointestinal surgery	Accuracy, precision, and AUC	LR, DT, gradient boosting, XGBC, and GBM	China

Quality Assessment

**Figure 2:**
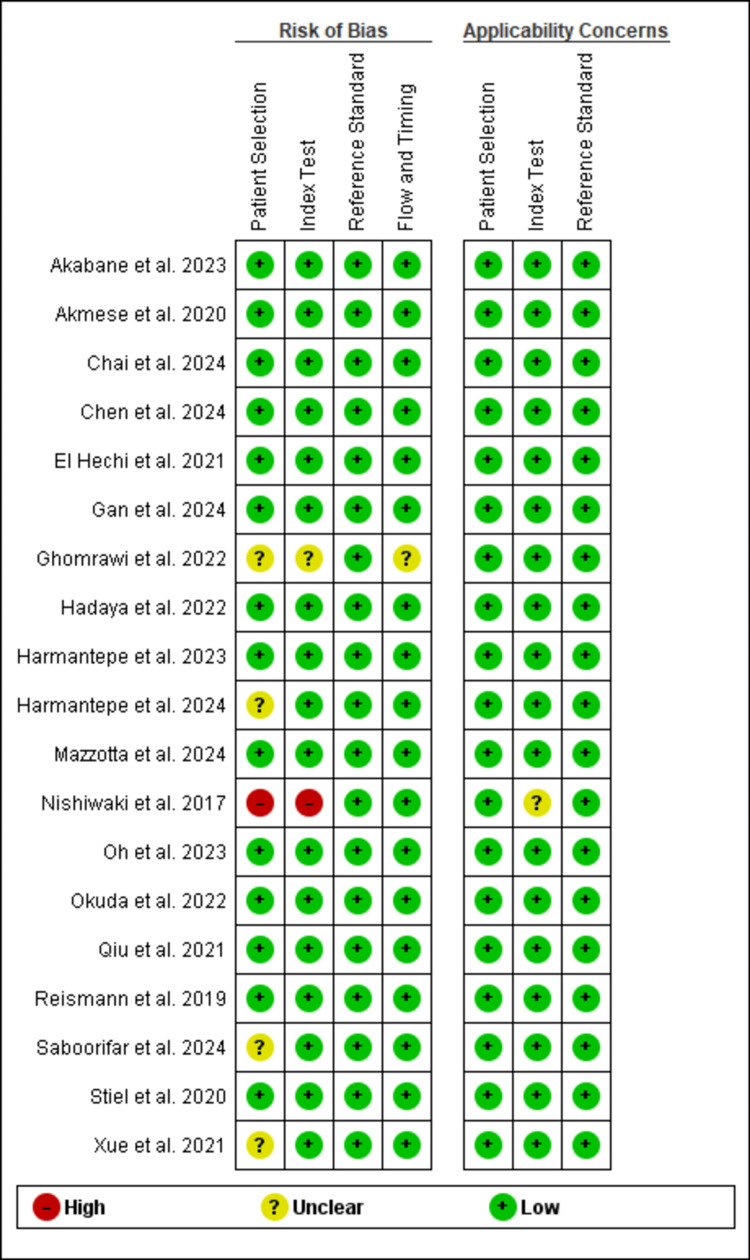
Quality assessment summary

**Figure 3:**
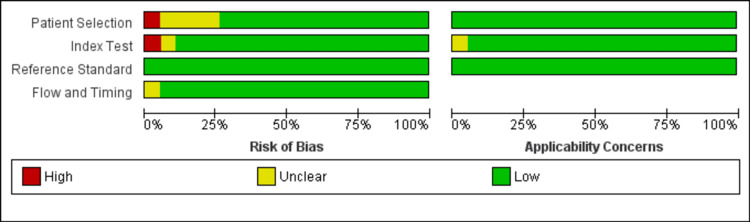
Methodological quality graph

The methodological quality of the included studies was generally high, with most studies demonstrating low risk of bias across all assessed domains, including patient selection, index test, reference standard, and flow and timing. Notable exceptions included Nishiwaki et al. [31], which showed a high risk of bias in patient selection and index test domains, and four studies with unclear risk of bias in patient selection due to insufficient reporting of selection criteria [19,27,34,36]. Applicability concerns were minimal, with nearly all studies (n=18; 95%) showing low concern across all domains, indicating good generalizability to clinical practice. Overall, the predominance of low-risk assessments suggests the evidence base is methodologically robust with minimal potential for bias affecting the validity of findings. All details are shown in Figure 2 and Figure 3.

Results

Appendicitis Diagnosis and Management

Akmese et al. 2020 [18]: The study aimed to develop and evaluate ML models to predict whether a patient needs appendix surgery by analyzing their blood test results at the hospital. The best model that was used in this study was called gradient boosted trees, which was able to predict with 95.31% accuracy whether a patient needed surgery or not. That model was able to give this precision just by checking blood test values, especially their neutrophil count, white blood cell count, and C-reactive protein levels. The approach would save time for doctors and give more accurate decisions about appendix surgery without needing to rely on computed tomography (CT) scans or other expensive imaging tests.

Ghomrawi et al. 2023 [19]: This study aimed to evaluate the ability of a consumer wearable smart activity tracker device (Fitbit) to detect abnormal recovery following pediatric appendectomy by analyzing physical activity, heart rate, and sleep data. The study found that ML models could accurately detect 83% of abnormal recovery days in complicated appendicitis and 70% in simple appendicitis. The findings supported the use of consumer wearables as monitoring tools for the early detection of post-surgical complications.

Harmantepe et al. 2023 [20]: This study aimed to predict acute appendicitis using ML algorithms with a simple and inexpensive method based on gender and hemogram data. The results showed that an ensemble model using (soft/hard) voting classification achieved the highest accuracy at 86.2% among the collected data, with a sensitivity of 83.7% and specificity of 88.6%. The study demonstrated that ML could effectively diagnose acute appendicitis using basic patient data, offering a practical, fast, and cost-effective diagnostic approach.

Reismann et al. 2019 [21]: This study developed and validated a method for automatic diagnosis of appendicitis using routine diagnostic parameters. The researchers analyzed patients' data, including full blood counts, C-reactive protein levels, and ultrasound-measured appendiceal diameters. The biomarker signature achieved 90% accuracy (93% sensitivity, 67% specificity) for diagnosing appendicitis and 51% accuracy (95% sensitivity, 33% specificity) for identifying complicated inflammation. The study showed that ML algorithms could effectively diagnose appendicitis using routine diagnostic parameters without requiring specialized imaging expertise.

Stiel et al. 2020 [22]: This study evaluated and improved different appendicitis scoring systems by analyzing 463 pediatric patients with suspected appendicitis across two centers. The researchers developed two new scoring approaches, namely, a modified Heidelberg appendicitis score and an AI-based score, which outperformed traditional scoring systems. The modified Heidelberg score achieved excellent results for uncomplicated appendicitis (positive predictive value (PPV) 95%, negative predictive value (NPV) 70%) and very good results for perforated appendicitis (PPV 34.4%, NPV 93.8%). The study demonstrated that simplified scoring systems incorporating fewer clinical features could provide superior diagnostic accuracy compared to more complex traditional scores.

Emergency Abdominal Surgery Risk Assessment and Prediction

Akabane et al. 2023 [23]: The study aimed to develop and evaluate ML models to predict in-hospital mortality for patients undergoing emergency colorectal surgery using clinical data from admission. The researchers compared four different models (logistic regression, random forests, gradient-boosting decision trees (GBDT), and multilayer perceptron), with GBDT showing the best performance compared to the other models. The developed model could help healthcare providers better evaluate mortality risk and manage resources for emergency colorectal surgery patients, which is especially important given that emergency procedures have significantly higher mortality rates (10-25%) than elective surgeries (1-3%).

Chen et al. 2024 [24]: This study developed ML models to predict perioperative risks for emergency abdominal surgery patients. The researchers compared five ML models (random forest, XGBoost, support vector machine, naive Bayes, and logistic regression) to predict five critical outcomes: postoperative mortality, pneumonia, surgical site infection, thrombosis, and mechanical ventilation >48 hours. The random forest model demonstrated superior performance across all outcomes, achieving the highest values and outperforming the other traditional models like the Emergency Surgery Score (ESS), Acute Physiology and Chronic Health Evaluation II (APACHE II), and American Society of Anesthesiologists (ASA) classification. Based on the obtained results, the research team developed a web-based calculator using the random forest model to provide real-time risk assessment, aiming to improve clinical decision-making and resource allocation in emergency surgery settings.

El Hechi et al. 2021 [25]: The researchers developed and validated the Predictive Optimal Trees in Emergency Surgery Risk (POTTER), which is an ML-based tool using optimal classification tree (OCT) methodology to predict 30-day outcomes for patients undergoing emergency general surgery. The researchers found that POTTER demonstrated exceptional predictive accuracy, with a c-statistic of 0.93 for mortality and 0.83 for morbidity across all patients. The researchers also found slightly lower performance in the emergency laparotomy subgroup. Unlike other ML models like the "black box", POTTER's decision tree approach allows physicians to understand how risk estimates are generated, making it a unique and valuable clinical decision support tool.

Hadaya et al. 2022 [26]: The study aimed to develop ML-based prediction models for respiratory failure patients, specifically following emergency general surgery operations. The study compared their performance to traditional regression models. The findings showed that the XGBoost model slightly outperformed logistic regression. Most importantly, XGBoost demonstrated excellent calibration across all risk levels. This study suggests that ML models could be more useful in clinical settings where probability predictions are needed.

Xue et al. 2021 [27]: The study aimed to investigate whether ML algorithms could predict pulmonary complications (PPCs) following emergency gastrointestinal surgery in patients with acute diffuse peritonitis. The research evaluated 926 cases using five different ML algorithms (logistic regression, decision tree, gradient boosting, XGBoost classifier (XGBC), and gradient boosting machine (GBM)). The findings revealed that 20.19% of patients developed PPCs, with the GBM model showing the best performance (area under the curve (AUC)=0.814; accuracy=80.6%). The study identified that key predictive factors include preoperative albumin, cholesterol levels, age, and platelet counts.

Acute Abdominal Pain and Triage

Chai et al. 2024 [28]: This study aimed to improve emergency department accuracy for patients with acute abdominal pain by developing ML models to predict surgical needs. The study compared four different ML models (light GBM, XGBoost, deep neural network, and random forest). Using routine triage data, including vital signs, physical examinations, and patient history, the light GBM model showed the best performance, outperforming the conventional logistic regression model. The main key predictive factors included physical examination findings, vital signs, physician risk assessments, and mode of arrival. The results demonstrate that ML models can enhance triage accuracy and help ensure critically ill patients receive timely surgical intervention.

Gan et al. 2024 [29]: In this study, the researchers aimed to develop an ML-based prediction model for triaging patients with acute abdominal pain to help healthcare professionals quickly identify critically ill patients and allocate medical resources efficiently. The researchers used logistic regression and multiple ML models to identify high-risk factors. They found that an ANN model demonstrated the most effective performance in predicting acute abdominal pain triage. The model achieved an impressive accuracy of 97.92%. The model depended on the history of diabetes and stroke, pulse, blood pressure, pale appearance, bowel sounds, and pain location to effectively predict the severity of acute abdominal pain, potentially providing a rapid and resource-efficient triage tool for emergency departments, especially in settings with limited medical resources.

Bowel Obstruction

Mazzotta et al. 2024 [30]: This study aimed to examine risk factors associated with major postoperative complications in patients undergoing surgery for bowel obstruction using ML approaches. The K-nearest neighbors (KNN) ML model achieved the best performance with an accuracy of 82% for predicting complications. KNN can also improve the perioperative management of patients with intestinal obstruction.

Nishiwaki et al. 2017 [31]: The aim was to develop a CNN model to detect small bowel strangulation using circulating cell-free DNA (ccfDNA) from blood samples. The study found that the length and concentration of ccfDNA could effectively distinguish between strangulated and non-strangulated bowel obstruction. Three ML models (support vector machine, decision tree, and random forest) were evaluated, with random forest achieving the best performance with perfect recall and a high accuracy of 94.7%.

Okuda et al. 2022 [32]: The goal was to develop an AI model to analyze surgical treatment decisions for intestinal obstruction in children using blood test data. Using a two-layer DL model, the model achieved 80.04% accuracy in predicting the need for surgery in the first layer and 66.78% accuracy in predicting intestinal necrosis in the second layer. The model demonstrated potential as a clinical decision support tool despite some limitations in necrosis prediction.

Qiu et al. 2021 [33]: This study aimed to develop a two-layer neural network model for predicting surgical treatment needs in childhood intestinal obstruction using routine blood counts and urine tests rather than medical imaging. The study analyzed children with intestinal obstruction. The first layer model achieved 80.04% accuracy in predicting surgery needs, while the second layer reached 70.4% accuracy in predicting complications. The study demonstrated that an ML approach using basic clinical data could effectively assist in surgical decision-making for pediatric intestinal obstruction cases.

Mesenteric Ischemia

Harmantepe et al. 2024 [34]: This research aimed to develop and validate an AI model using ML to predict hospital mortality in patients with acute mesenteric ischemia. The study found that the support vector classifier (SVC) and voting classifier (VC) showed the best performance, both achieving a similar high accuracy (84%). The SVC demonstrated high effectiveness with a high sensitivity of 100%, a specificity of 77%, and an AUC of 90%, providing an efficient and rapid method for predicting mortality in these cases.

Cholecystitis and Gallbladder

Oh et al. 2023 [35]: This study compared DL with experienced physicians in diagnosing gangrenous cholecystitis using CT images. The DL model outperformed the human reviewers, achieving better diagnostic accuracy (72.6%), sensitivity (72.6%), and specificity (86.3%). The study demonstrated that AI could effectively assist in identifying high-risk cases requiring emergency surgery.

Saboorifar et al. 2024 [36]: The study aimed to assess the diagnostic performance of a support vector machine algorithm for acute cholecystitis in emergency department settings. The findings showed that among included patients, the used model resulted in promising results with a sensitivity of 83.08%, a specificity of 80.21%, an accuracy of 81.37%, and an AUC of 0.89. The model relied on key predictive variables like preoperative albumin, cholesterol levels, and platelet counts, demonstrating potential as a diagnostic tool, though requiring further validation for clinical implementation.

Discussion

This systematic review revealed significant findings across six emergency surgery categories, demonstrating the effectiveness of AI applications. In appendicitis management, gradient-boosted trees achieved the highest accuracy (95.31%) using basic blood tests, while consumer wearables showed promise in detecting post-surgical complications with 70-83% accuracy. For emergency abdominal surgery risk assessment, the POTTER tool demonstrated exceptional predictive accuracy (c-statistic: 0.93 for mortality, 0.83 for morbidity), and random forest models consistently outperformed traditional risk assessment tools, including in predicting pulmonary complications (80.6% accuracy). Acute abdominal pain triage saw particularly impressive results, with an ANN achieving 97.92% accuracy, while light GBM models outperformed conventional methods using routine triage data. In bowel obstruction cases, random forest models reached 94.7% accuracy for strangulation detection, two-layer models showed 80% accuracy for surgical needs prediction, and KNN achieved 82% accuracy for complication prediction. For mesenteric ischemia, SVCs demonstrated 84% accuracy with high sensitivity (100%) and specificity (77%) in mortality prediction. Finally, DL outperformed human reviewers in cholecystitis diagnostics with 72.6% accuracy, and support vector machines showed promising results with 81.37% accuracy. Across all categories, AI models consistently demonstrated high accuracy using basic clinical data, often surpassing traditional diagnostic and predictive methods.

While patient history, physical examination, laboratory results, and scoring systems are important for the diagnosis of appendicitis, imaging studies have proven most effective in confirming suspected acute appendicitis. Among imaging techniques, ultrasound measurement of the appendix diameter has emerged as a particularly valuable diagnostic indicator [37,38].

The AI models have been developed to enhance surgical decision-making for patients with suspected acute appendicitis, which will result in the prevention of unnecessary surgical procedures and their associated complications [18]. While increased CT usage before surgery has helped reduce incorrect appendix removals, the AI model offers an alternative diagnostic pathway with high efficiency [21].

The significant disparity in mortality rates between elective (1-3%) and emergency colorectal surgeries (12-20%) highlights the critical need for accurate risk assessment and prediction tools in emergency abdominal surgery [39]. This remarkable difference underscores the complexity of emergency surgical decision-making and emphasizes the importance of developing reliable predictive models for better patient outcomes. AI usage could show a great benefit in the prediction of in-hospital mortality after emergency colorectal surgery, which will result in improving patient outcomes and be life-saving [23,24]. On the other hand, an orthopedic study demonstrates that conventional knee arthroplasty actually outperforms robotic-assisted surgery in several key parameters, including operative time and clinical outcomes [40].

These findings highlight the importance of context-specific evaluation when implementing new medical technologies rather than assuming technological advancement always yields superior results.

The systematic review demonstrates several notable strengths, as this study covers the most recent improvements and developments in the field of AI and robotic-assisted emergency. We covered different aspects of the different present studies as we categorized the included studies into six different domains to show the effects within each section. However, this review faced certain limitations. All included studies were based on retrospective designs, which are inherently prone to bias. Furthermore, there was significant variation in the reporting of performance metrics, as the estimation tools and evaluation methods differed across studies. This lack of standardization, combined with potential heterogeneity in the study populations, may have impacted the comparability and generalizability of the findings. A single study comparing all the different types of available AI systems in a direct comparison would result in more reliable results on the best AI in each section. Future research should include comprehensive cost-effectiveness analyses of AI implementation in surgical settings to identify optimal systems that are both clinically effective and economically feasible for widespread adoption in healthcare facilities.

## Conclusions

This systematic review demonstrates the significant potential of AI and ML applications across various domains of emergency surgery. From appendicitis management to cholecystitis diagnostics, AI models consistently showed high-performance metrics, with accuracy rates ranging from 72% to 98%. High accuracy rates were found when estimating acute abdominal pain triage and diagnosing appendicitis. It should be taken into consideration that the results in abdominal surgery risk assessment, bowel obstruction prediction, mesenteric ischemia mortality prediction, and cholecystitis diagnosis were promising and showed advantages over conventional methods. These findings indicate that AI would enhance clinical decision-making in emergency surgical settings.

## References

[1] Long D, Magerko BS (2020). What is AI literacy? Competencies and design considerations. CHI '20: Proceedings of the 2020 CHI Conference on Human Factors in Computing Systems.

[2] Ahmad Z, Rahim S, Zubair M, Abdul-Ghafar J (2021). Artificial intelligence (AI) in medicine, current applications and future role with special emphasis on its potential and promise in pathology: present and future impact, obstacles including costs and acceptance among pathologists, practical and philosophical considerations. A comprehensive review. Diagn Pathol.

[3] El Naqa I, Murphy MJ (2015). What is machine learning?. Machine Learning in Radiation Oncology.

[4] Bishop CM (2006). Pattern Recognition and Machine Learning. https://link.springer.com/book/9780387310732.

[5] Bini SA (2018). Artificial intelligence, machine learning, deep learning, and cognitive computing: what do these terms mean and how will they impact health care?. J Arthroplasty.

[6] Greenhill AT, Edmunds BR (2020). A primer of artificial intelligence in medicine. Tech Innov Gastrointest Endosc.

[7] Zou J, Han Y, So SS (2008). Overview of artificial neural networks. Methods Mol Biol.

[8] Indolia S, Goswami AK, Mishra SP, Asopa P (2018). Conceptual understanding of convolutional neural network- a deep learning approach. Procedia Comput Sci.

[9] Hirani R, Noruzi K, Khuram H (2024). Artificial intelligence and healthcare: a journey through history, present innovations, and future possibilities. Life (Basel).

[10] Kaul V, Enslin S, Gross SA (2020). History of artificial intelligence in medicine. Gastrointest Endosc.

[11] Kulikowski CA (2019). Beginnings of artificial intelligence in medicine (AIM): computational artifice assisting scientific inquiry and clinical art - with reflections on present aim challenges. Yearb Med Inform.

[12] Shortliffe EH (1977). Mycin: a knowledge-based computer program applied to infectious diseases. Proc Annu Symp Comput Appl Med Care.

[13] Lo SB, Lou SA, Lin JS, Freedman MT, Chien MV, Mun SK (1995). Artificial convolution neural network techniques and applications for lung nodule detection. IEEE Trans Med Imaging.

[14] Bakkar N, Kovalik T, Lorenzini I (2018). Artificial intelligence in neurodegenerative disease research: use of IBM Watson to identify additional RNA-binding proteins altered in amyotrophic lateral sclerosis. Acta Neuropathol.

[15] Mintz Y, Brodie R (2019). Introduction to artificial intelligence in medicine. Minim Invasive Ther Allied Technol.

[16] Ghazal AH, Fozo ZA, Matar SG, Kamal I, Gamal MH, Ragab KM (2023). Robotic versus conventional unicompartmental knee surgery: a comprehensive systematic review and meta-analysis. Cureus.

[17] Page MJ, McKenzie JE, Bossuyt PM (2021). The PRISMA 2020 statement: an updated guideline for reporting systematic reviews. BMJ.

[18] Akmese OF, Dogan G, Kor H, Erbay H, Demir E (2020). The use of machine learning approaches for the diagnosis of acute appendicitis. Emerg Med Int.

[19] Ghomrawi HM, O'Brien MK, Carter M (2023). Applying machine learning to consumer wearable data for the early detection of complications after pediatric appendectomy. NPJ Digit Med.

[20] Harmantepe AT, Dikicier E, Gönüllü E, Ozdemir K, Kamburoğlu MB, Yigit M (2023). A different way to diagnosis acute appendicitis: machine learning. Pol Przegl Chir.

[21] Reismann J, Romualdi A, Kiss N, Minderjahn MI, Kallarackal J, Schad M, Reismann M (2019). Diagnosis and classification of pediatric acute appendicitis by artificial intelligence methods: an investigator-independent approach. PLoS One.

[22] Stiel C, Elrod J, Klinke M (2020). The modified Heidelberg and the AI appendicitis score are superior to current scores in predicting appendicitis in children: a two-center cohort study. Front Pediatr.

[23] Akabane S, Miyake K, Iwagami M, Tanabe K, Takagi T (2023). Machine learning-based prediction of postoperative mortality in emergency colorectal surgery: a retrospective, multicenter cohort study using Tokushukai medical database. Heliyon.

[24] Chen B, Sheng W, Wu Z (2024). Machine learning based peri-surgical risk calculator for abdominal related emergency general surgery: a multicenter retrospective study. Int J Surg.

[25] El Hechi MW, Maurer LR, Levine J (2021). Validation of the artificial intelligence-based Predictive Optimal Trees in Emergency Surgery Risk (POTTER) calculator in emergency general surgery and emergency laparotomy patients. J Am Coll Surg.

[26] Hadaya J, Verma A, Sanaiha Y, Ramezani R, Qadir N, Benharash P (2022). Machine learning-based modeling of acute respiratory failure following emergency general surgery operations. PLoS One.

[27] Xue Q, Wen D, Ji MH, Tong J, Yang JJ, Zhou CM (2021). Developing machine learning algorithms to predict pulmonary complications after emergency gastrointestinal surgery. Front Med (Lausanne).

[28] Chai C, Peng SZ, Zhang R, Li CW, Zhao Y (2024). Advancing emergency department triage prediction with machine learning to optimize triage for abdominal pain surgery patients. Surg Innov.

[29] Gan T, Liu X, Liu R (2024). Machine learning based prediction models for analyzing risk factors in patients with acute abdominal pain: a retrospective study. Front Med (Lausanne).

[30] Mazzotta AD, Burti E, Causio FA (2024). Machine learning approaches for the prediction of postoperative major complications in patients undergoing surgery for bowel obstruction. J Pers Med.

[31] Nishiwaki K, Yamada T, Iwai T, Takahashi G, Uchida E, Ohwada H (2017). Detecting small bowel strangulation using circulating cell-free DNA with machine learning. Int J Mach Learn Comput.

[32] Okuda Y, Saida T, Morinaga K (2022). Diagnosing gangrenous cholecystitis on computed tomography using deep learning: a preliminary study. Acute Med Surg.

[33] Qiu WR, Chen G, Wu J, Lei J, Xu L, Zhang SH (2021). Analyzing surgical treatment of intestinal obstruction in children with artificial intelligence. Comput Math Methods Med.

[34] Harmantepe AT, Dulger UC, Gonullu E, Dikicier E, Şentürk A, Eröz E (2024). A method for predicting mortality in acute mesenteric ischemia: machine learning. Ulus Travma Acil Cerrahi Derg.

[35] Oh S, Ryu J, Shin HJ, Song JH, Son SY, Hur H, Han SU (2023). Deep learning using computed tomography to identify high-risk patients for acute small bowel obstruction: development and validation of a prediction model : a retrospective cohort study. Int J Surg.

[36] Saboorifar H, Rahimi M, Babaahmadi P, Farokhzadeh A, Behjat M, Tarokhian A (2024). Acute cholecystitis diagnosis in the emergency department: an artificial intelligence-based approach. Langenbecks Arch Surg.

[37] Coyne SM, Zhang B, Trout AT (2014). Does appendiceal diameter change with age? A sonographic study. AJR Am J Roentgenol.

[38] Benabbas R, Hanna M, Shah J, Sinert R (2017). Diagnostic accuracy of history, physical examination, laboratory tests, and point-of-care ultrasound for pediatric acute appendicitis in the emergency department: a systematic review and meta-analysis. Acad Emerg Med.

[39] Krajewski S, Brown J, Phang PT, Raval M, Brown CJ (2011). Impact of computed tomography of the abdomen on clinical outcomes in patients with acute right lower quadrant pain: a meta-analysis. Can J Surg.

[40] Fozo ZA, Ghazal AH, Hesham Gamal M, Matar SG, Kamal I, Ragab KM (2023). A systematic review and meta-analysis of conventional versus robotic-assisted total knee arthroplasty. Cureus.

